# Assessment of Thiopurine–based drugs according to Thiopurine S-methyltransferase genotype in patients with Acute Lymphoblastic Leukemia

**Published:** 2014-03-15

**Authors:** F Azimi, M Jafariyan, S Khatami, Y Mortazavi, M Azad

**Affiliations:** 1 M.S.c student of medical genetics, Zanjan University of Medical Science, Zanjan,Iran; 2 PhD student of molecular medicine, Zanjan University of Medical Science, Zanjan,Iran; 3 Assistant professor in hematology, Zanjan University of Medical Science, Zanjan,Iran; 4 Assistant professor in hematology, Ghazvin University of Medical Science, Ghazvin,Iran

**Keywords:** Acute lymphoblastic leukemia, Azathioprine, 6-Mercaptopurine, 6-Thioguanine, Myelosuppression, Polymorphism

## Abstract

For the past half century, thiopurines have earned themselves a reputation as effective anti-cancer and immunosuppressive drugs.

Thiopurine S-methyltransferase (TPMT) is involved in the metabolism of all thiopurines and is one of the main enzymes that inactivates mercaptopurine. 6-MP is now used as a combination therapies for maintenance therapy of children with acute lymphocytic leukemia (ALL). In all patients receiving mercaptopurine, there is a risk of bone marrow suppression.

TPMT activity is inherited as a monogenic, co-dominant trait. More than 25 variants are known. Genetic testing is available for several TPMT variant alleles. Most commonly TPMT*2, *3A, and *3C are tested for, which account for >90% of inactivating alleles. Differences in DNA that alter the expression or function of proteins that are targeted by drugs can

contribute significantly to variation in the responses of individuals.Genotyping may become part of routine investigations to help clinicians tailor drug treatment effectively. This success is mainly due to the development of combination therapies and stratification of patients according to risk of treatment failure and relapse, rather than the discovery of new drugs. The aim of this study was to investigate the effect of genotype or methyltransferase enzyme activity before starting therapy in children with ALL. This can prevent the side effect of thiopurine drugs. In fact, the common polymorphism of this enzyme in population could be a prognostic factor in relation to drug use and treatment of patients with ALL.

## Introduction

Since the introduction of combination chemotherapy for the treatment of acute lymphoblastic leukemia (ALL) in children, the prognosis for this disease has improved steadily such that most trials now report a long-term survival rate of over 70%. However, current treatment protocols are complex and are associated with signiﬁcant toxicity [1].

There are therefore two major priorities in the treatment of childhood ALL; further improvement in the survival rate and a reduction in treatment-associated toxic side effects. One method, which may be used to achieve these goals, is the more effective use of currently available chemotherapy. At present, most patients are given cytotoxic drugs at a dosage determined by their surface area, despite the fact that, for several of the agents used, there is good evidence that this results in marked differences in the amount of drug whichreaches its target [2]. This is exempliﬁed by the thiopurine, 6-mercaptopurine (6-MP), used in many regimes as part of themaintenance phase of therapy.

The level of expression of the enzyme thiopurine methyltransferase (TPMT) is an important determinant of the metabolism of thiopurines used in the treatment of acute lymphoblastic leukemia (ALL).

Thiopurine drugs (azathiopurine, 6-mercaptopurine, thioguanine) are cytotoxic agents increasingly used to treat a variety of conditions including acute lymphoblastic leukemia, inflammatory bowel disease, Crohn's disease, and multiple sclerosis.

The importance of TPMT activity in the inactivation of 6-MP is demonstrated by the severe myelosuppression suffered by individuals with a congenital absence of the enzyme when given standard doses of 6-MP or the closely related drug, azathioprine.

6-MP is the common component of regimen for intensiﬁcation and consolidation as well as long-term continuation therapy of childhood ALL. 6-TG on the other hand is commonly applied for blocks of intensive treatment, and was initially developed as acomponent of induction and remission maintenance therapy of myeloid leukemias. The routine dose of 6-MP employed for chemotherapeutic maintenance of ALL is 75 mg/m2 of body surface area [3].

**Metabolism of thiopurines**

Mercaptopurine is inactivated by two different pathways, one being catalyzed by TPMT.

Individuals who inherit two nonfunctional TPMT alleles (~1 in 178 to 1 in 3,736) experience life-threatening myelosuppression, due to high levels of TGNs, if they receive conventional doses of mercaptopurine. Individuals who are heterozygous for nonfunctional TPMT alleles (~3–14%) are at asignificantly higher risk for toxicity than individuals with two functional alleles. However, some of these individuals, ~40–70%, can tolerate the full dose of mercaptopurine. This maybe because heterozygous-deficient individuals have lower concentrations of less active metabolites, such as MeMPN (methylmercaptopurine nucleotides), than homozygous-deficient individuals [3,4,5,6].

Another mercaptopurine inactivation pathway is oxidation, which is catalyzed by xanthine dehydrogenase, XDH (also known as xanthine oxidase). If this pathway is inhibited, catabolism of mercaptopurine is reduced leading to mercaptopurine toxicity. Therefore, a lower dose of mercaptopurine is required in patients taking the XDH inhibitor, allopurinol .

**Figure 1:**
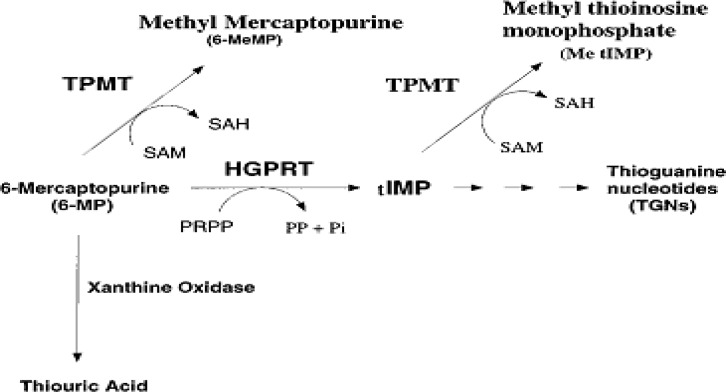
The metabolism of 6-mercaptopurine

6-MP is a prodrug, which requires activation before it can exert its cytotoxic effects. As an analogue of hypoxanthine, it can act as a substrate for hypoxanthine guanine phosphoribosyl-transferase (HGPRT) leading to the formation of 6-thioinosinemonophosphate (TIMP). Sequential reactions involving inosinemonophosphate dehydrogenase and guanine monophosphatesynthetase yields thioguanosine monophosphate (TGMP). Sequential reactions by phosphokinases yields 6-thioguanosinediphosphate and triphosphate (TGDP and TGTP). TGMP, TGDP, and TGTP are collectively termed thioguanine nucleotides (TGNs, see Figure 1). Incorporation of 6-TGTP into DNA isbelieved to trigger cell death, probably by a process thatinvolves the mismatch repair pathway [7,8].

**Thiopurinemethyltransferase (TPMT)**

Thiopurine methyltransferase (TPMT) is a cytosolic methylating enzyme whose physiologicalrole, despite extensive investigations remains unclear. However,this enzyme is known to catalyze S-methylation of aromatic and heterocyclic compounds, preferentially thio compounds such as 6-MP and 6-TG. 

It has a molecular mass of 26 kDa and is expressed inthe liver, kidneys, intestine, erythrocytes, leukocytes and a numberof other tissues [9]. The discovery that levels of TPMT activity in human tissues areinﬂuenced by a common genetic polymorphism represents themost important example of the inﬂuence of pharmacogenetics on anti-cancer therapy as one of the best examples of the potential importance of pharmacogenetics for clinical medicine ingeneral [10]. Speciﬁcally, it is nowknown that a reduction in TPMT activity, caused by genetic polymorphism, results in severe and sometimes fatal haematological toxicity in patients treated with standard doses of thiopurines such that the dose must bedecreased for patients with heterozygous or homozygous polymorphisms in the TPMT gene. Conversely, patients with very high TPMT activity may be undertreated.Numerous genetic polymorphisms have been identiﬁed which are or may be associated with decreased levels of TPMT enzyme activity and/or enhanced toxicity of thiopurines [11].

This polymorphisms are distributed throughout the protein structure they all are able to exert an effect on the active site. The frequency of distribution of erythrocyte TPMT activity in 298 control subjects was found to be trimodal. Approximately 1 in 300 individuals (0.3%) had low orundetectable levels of TPMT activity, with intermediate levels inapproximately 10% of individuals [10].

Variant human alleles of TPMT proven to be associated with decreased catalytic activity can involve point mutations in the open-reading frame or at intron/exon splice sites. Patients that inherit one wild-type allele and one of the mutant alleles have intermediate TPMT activity while those that are homozygous for mutant alleles are TPMT deﬁcient . Other alterations detected in the TPMT gene include deletion of exons six and nine and polymorphisms in the variable number of tandem repeats [11,12,13].

Four alleles (TPMT*2, *3A, *3B, and *3C) account for ∼95% of inherited TPMT deficiency and have been biochemically characterized [6,14,15,16].

The *3A allele results in an∼400- fold decrease in protein levels and no detectable enzyme activity. The TPMT*3B allele results in a four fold decrease in protein levels. The TPMT*2 allele contains an alanine to proline substitution at residue 80 that results in ∼100-fold decrease in TPMT activity and very low levels of immunological protein and the TPMT*3C allele results in a 1.4-fold reduction in protein levels [17,18].

Several of these alleles cause rapid protein degradation and/or aggregation, making it extremely difficult to study the structural impact of the TPMT polymorphisms experimentally [19].

The TPMT gene encodes one of the main enzymes involved in the metabolism of thiopurines, such as mercaptopurine. TPMT activity is inherited as a monogenic, co-dominant trait. TPMT is highly polymorphic—more than 25 variants are known [20]. 

• TPMT*2 (238G>C)

• TPMT*3A (contains two SNPs, *3B and *3C)

• TPMT*3B (460G>A)

• TPMT*3C (719A>G)

The wild-type allele, TPMT*1, encodes the fully active enzyme; while TPMT*2, TPMT*3A and TPMT*3C are the most prevalent in Caucasians, accounting together 80 to 95% of the polymorphic alleles that lead to a signiﬁcantreduction in enzyme activity due to enhanced rates of proteolysis the mutant proteins [21]. 

The frequency of TPMT alleles varies among different populations. In the United States, the

most common low-activity allele in the Caucasian population is TPMT*3A (~5%). This alleleis also found in individuals who originate from India and Pakistan, but less frequently [22,23,24].

In East Asian, African-American, and some African populations, the most common variant is TPMT*3C (~2%), although TPMT*8 may be more common in African populations than previously thought (~2%). In general, TPMT*2 occurs much less commonly, and TPMT*3B occurs rarely [23,25].

Population studies in Caucasian, East and West African, African–American, Chinese, Japanese and Southwest Asian populations have demonstrated the utility of this approach [21,26,27,28].

However, the frequency and pattern of mutant TPMT alleles is different among various ethnic populations. For example, Southwest Asians (Indian, Pakistani) have a lower frequency of mutant TPMT alleles and all mutant alleles identiﬁed to date are TPMT*3A [21].

This is in contrast with the East and West African population in which the frequency of mutantalleles is similar to Caucasians, but all mutant alleles in the African populations are TPMT*3C [27,28].

Among African–Americans, TPMT*3C is the most prevalent, but TPMT*2 and TPMT*3A are also found, reﬂecting the integration of Caucasians and African–Americans genes in the US populations [26].

Mehdi Azad and et al have shown in Iranian Population a prevalence of TPMT*2 was 7.08%. TPMT*3C and *3A were found in 2.47% and 2.18% of the samples, respectively. TPMT*3B variant was not detected in Iranian subjects. These results can help to organize national pretreatment strategies in patients with acute lymphoblastic leukemia (ALL) or other diseases requiring thiopurine medication in their standard therapy [29]. In other study, Mehdi Azad and et al have investigated the frequency of the most prevalent Thiopurine S-Methyl Trasferase alleles in referrals to Shariati Hospital in Tehran, and showed Mutant TPMT alleles were found in 11.8% of subjects (15 out of 127). Nine had TPMT*2, 4 TPMT *3C and 2 TPMT*3A. The data showed the necessity of TPMT polymorphisms assessment before administration of thiopurine drugs [30].

Pharmacokinetic and pharmacodynamic studies of 6-MP and 6-TG in TPMT knockout mice with high, medium and no TPMT activity indicated that 6-MP was signiﬁcantly more affected by TPMT polymorphisms than 6-TG [31]. Those studies corroborated earlier research by Dervieux et al [32] who found that elevation of TPMT activity in human CCRF-CEM cell lines by retroviral genetransfer rendered the cells less sensitive to 6-TG, but more sensitive to 6-MP. This effect was also seen in another study by Coulthard et al. [9] in which human embryonic kidney cells were transfected with TPMT cDNA under the control of an induciblesysteminwhich they showed a 4.4-fold increase in sensitivity to 6-MP and a 1.6-fold decrease in sensitivity to 6-TG. 

TPMT activity is inversely related to TGN concentrations in theerythrocytes of children treated for leukemia [33]. High erythrocyte concentrations of TGNs are correlated with the degree ofleucopenia and a good prognosis [34] whereas low concentrations are associated with an increased risk for relapse [35].

 Several recent studies have identiﬁed an interaction between 6-MP pharmacology and the incidence of secondary malignancies, including brain tumors after radiotherapy. At ﬁrst theresults appear to be conﬂicting, however, these anomalies aremostlikely to be protocol dependent. Essentially, for patients treatedwith the St. Jude Children’s Hospital protocols, low TPMT activity is associated with higher risk ofsecondary cancers, in contrast to patients treated with the BFM protocols where no such association was found [36].

**Genetic Testing**

Genetic testing is available for several TPMT variant alleles. Most commonly TPMT*2, *3A, and *3C are tested for, which account for >90% of inactivating alleles. Rare or previously undiscovered variants will not be detected by variant-specific genotyping methods [37].

Phenotype testing is also available. For example, the TPMT activity in red blood cells can be measured directly. However, the results will not be accurate in patients who have received recent blood transfusions [38]. Method for the measurement of mercaptopurine metabolites (TGN and MeMPN) are also available.

**Therapeutic Recommendations**

**FDA statement**

If a patient has clinical or laboratory evidence of severe toxicity, particularly myelosuppression, TPMT testing should be considered. Substantial dose reductions of mercaptopurine are generally required for homozygous-TPMT deficiency patients (two non-functional alleles) to avoid the development of life threatening bone marrow suppression.

Although heterozygous patients with intermediate TPMT activity may have increased mercaptopurine toxicity, this is variable, and the majority of patients tolerate normal doses of mercaptopurine [38].

**CPIC statement**

Testing for TPMT status is recommended prior to starting mercaptopurine therapy, so that the starting dosages can be adjusted accordingly (see Table 1 for dosing recommendations). In homozygous variant individuals, consider an alternative agent for nonmalignant conditions and drastically reduce doses in malignant conditions. In heterozygousindividuals, depending on the disease being treated, starting doses should be reduced. In both patient groups, a longer period of time should be left after each dose adjustment to allow for asteady state to be reached [6]. 

Most population studies of TPMT activity have used RBC asa convenient source of samples. Levels in RBC have beenshown to reﬂect levels in other tissues, including lymphoblasts [39].

 One major problem with the use of RBC is the fact thatsamples must be obtained before transfusion. Previous reports in which levels of TPMT activity in RBC increaseduring maintenance therapy also suggests that epigenetic factors may affect the rate of transcription [40].

## Results

During the time we have witnessed dramatic improvement in survival rates of leukemia patients. This success is mainly due to the development of combination therapies and stratiﬁcation of patients according to risk of treatment failure and relapse, rather than the discovery of new drugs.

Studies of pharmacogenetics designed to improve drug safety and efﬁcacy have been already proven to be advantageous in the case of the inﬂuence of genetic polymorphisms in the TPMT gene on response to thiopurines. 

Gene array technology should aid researchers in ﬁnding new targets for thiopurines and may help predict the response to these agents through comparison of the expression proﬁles of responsive and non-responsive patients. Application of HapMap tools or specially designed biochips to genotype TPMT already show great promise and could bemodiﬁed to determine other important factors affecting thiopurine efﬁcacy [41].

Although studies of the role of TPMT in determining the response of patients to continuing therapy have concentrated on the effects of this enzyme on drug conversionwithin leukemic cells, it is possible that variations in expression in the liver and intestine will also inﬂuence response through an effect on systemic pharmacokinetics. Family studies have shown that TPMT deﬁciency is inheritedas an autosomal recessive trait [42].

There is also emerging evidence that these drugs are responsible for changes in methylation levels within the cells. The thiopurine drugs 6-MP, and meMRP caused a reduction in DNA methylation in the T-cell acute lymphoblastic leukemic cell line MOLT-4 [43]. Hogarth et al. [42] have shown that there is a decrease in demethyltransferase (DNMT) activity and protein after thiopurine treatment which was inﬂuenced by TPMT for 6-TG, but was not inﬂuenced 6-MP in human embryonic kidney cells. As demethylating agents are known to be active in leukemia, it is possible that inhibition of DNA methylation by the thiopurine drugs may also contribute to their cytotoxic affects.

Ultimately, stratiﬁcation of patients according to their general pharmacogenetic characteristics through application of expressionproﬁling techniques could further improve treatments outcomesby providing individually tailored combination therapy. 

**Table I:** TPMT phenotypes and the therapeutic recommendations for mercaptopurine therapy [6]

**Phenotype**	**Phenotype details**	**Genotype**	**Examples of diplotypes**	**Therapeutic recommendations for mercaptopurine**	
Homozygous wildtype(“normal”)	High enzyme activity. Found in ~86-–97% of patients.	Two or more functional alleles	*1/*1		Start with normal starting dose. Adjust doses of mercaptopurine along with concomitant medications. Allow 2 weeks to reach steady state after each dose adjustment.
Heterozygous	Intermediate enzyme activity. Found in ~3— 14% of patients.	One functional allele plus one nonfunctional allele	*1/*2 *1/*3A *1/*3B *1/*3C *1/*4		Start with reduced doses (30–70% of the full dose). Adjust doses of mercaptopurine depending on degree of myelosuppression and disease- specific guidelines. Allow 2–4 weeks to reach steady state after each dose adjustment
Homozygous and disease- specific guidelines. Allow 4–6 weeks to reach steady state after each dose adjustment	variant Low or deficient enzyme activity. Found in ~1 in 178 to 1~3736 patients.	Two nonfunctional alleles	*3A/*3A *2/*3A *3C/*3A *3C/*4 *3C/*2 *3A/*4		Consider alternative agents for nonmalignant conditions. For malignancy, start with drastically reduced doses (reduce daily dose by 10-fold and dose thrice weekly instead of daily)Adjust doses ofmercaptopurine based on degree ofmyelosuppression
